# Early nocturnal meal skipping alters the peripheral clock and increases lipogenesis in mice

**DOI:** 10.1186/1743-7075-9-78

**Published:** 2012-09-10

**Authors:** Chika Yoshida, Nahoko Shikata, Shinobu Seki, Naoto Koyama, Yasushi Noguchi

**Affiliations:** 1 Institute for Innovation, Ajinomoto Co. Inc, 1-1 Suzuki-Cho, Kawasaki-ku, Kawasaki, 210-8681, Japan

**Keywords:** Circadian rhythm, Lipogenesis, Obesity, Meal skipping, Clock, Night eating syndrome

## Abstract

**Background:**

In humans, skipping meals, especially breakfast, has been associated with obesity and other related syndromes. Recent studies in rodents suggest that fasting and feeding times are potential factors that affect the peripheral circadian clocks and metabolism. However, the link between fasting and obesity in rodents has yet to be fully demonstrated.

**Method:**

We conducted early nocturnal fasting (ENF) from zeitgeber time (ZT) 12 to 18 for 4 consecutive days in C57B6 mice. The first set of experiments was performed under *ad libitum* conditions, where ENF and free-feeding (FF) control groups were compared. The second set was performed under isocaloric adjustment by restricting the diet to 90% of the basal intake of ENF mice. Calorie-restricted ENF (ENF-CR) mice were then compared with isocaloric controls (IC-control). Body weight, food intake, core body temperature, activity, adiposity, and clock-related gene expression levels in the liver and adipose tissues were investigated. A stable isotopic analysis was also conducted to estimate *de novo* lipogenesis fluxes.

**Results:**

In the *ad libitum* condition, the ENF mice ate more during the day, increased their overall daily food intake and gained more weight than FF-control mice. The amplitude of the body core temperature rhythm in ENF mice was also lower than in the FF-controls. Under isocaloric conditions, ENF-CR attenuated the CR-induced body weight loss, compared with the IC-control. ENF-CR also altered the acrophase time of the expression of the clock genes, which is associated with time-shift of genes involved in lipid metabolism and increased lipogenesis, compared with the IC-control.

**Conclusions:**

ENF in nocturnal mice disturbs the peripheral clock and increases *de novo* lipid synthesis and results in a predisposition to obesity.

## Introduction

A number of important biochemical, behavioral and physiological phenomena are under the control of a circadian rhythm [1,2]. Feeding behavior, in terms of its timing and periodicity, in addition to nutritional quality, has a significant impact on circadian rhythms and metabolism. For example, diet-induced thermogenesis is known to show circadian variation, with the highest occurrence in the morning and the lowest in the evening in humans [3]. *De novo* lipid synthesis increases during the time period of taking meals and then drops during the rest period [4,5]. Recent human epidemiological studies suggest that skipping the first meal of the day increases the chances of developing obesity and related metabolic failures [6-10]. Nocturnal eating, especially night eating syndrome (NES), could be considered to be an abnormality in the circadian rhythm of meal timing and sleep onset and is strongly related to excess weight gain and obesity [11]. Along with such observations in humans and animal studies that investigate circadian rhythms have been conducted at the behavioral, physiological, and more recently, molecular levels [12-16]. In mammals, the central clock is located in the suprachiasmatic nucleus (SCN) of the anterior hypothalamus in the brain [17,18]. Recent studies also show that circadian machinery exists in most peripheral tissues and responds differently to dietary cues [19-22]. Having an irregular feeding schedule is reported to cause decoupling between the peripheral and SCN circadian oscillators [23,24]. Nutrient such as lipid or glucose absorption also shows circadian rhythm, and the expressions of proteins related to nutrient absorption are effected by time-restricted feeding [20]. Among the peripheral tissues, the liver is considered to be one of the organs that is most sensitive to feeding conditions. For example, restricted feeding is reported to affect the circadian periodicity of rat hepatic *Per1* expression [25,26], and the hepatic *Per2* gene in mice is affected by both the starvation interval and the food amount [27]. Such food-induced periodical shifts proceed faster in the liver than in the kidney, heart, or pancreas in mice [23].

These findings in both humans and animals suggest that the timing of food intake itself could be related to weight gain *via* alteration of the peripheral clock and metabolism. Recent studies show that diurnal high-fat feeding leads to greater weight gain than normal nocturnal feeding in mice [28]. Likewise, limiting the feeding time to the nocturnal period prevents a body weight increase and reverts metabolic disturbances in a rat shift work model [29]. This result shows that even in the situation that the animal are subject to be active in the light (“rest”) period, it is better to take in food during its innate “active” period for prevention of life-style related diseases. However, the information about the difference during the “active” period is few. Recent study in rodents showed that time of meal during the “active” period and its dietary quality influence metabolic parameters [30], though, the molecular or metabolic changes caused by fasting in the “active” phase have not yet been examined. In human, for example, skipping breakfast means they are fasted at the beginning of the day. Thus, it is one of the cases of fasting in “active” phase. Taking in food at the onset of active phase plays important roles and not having breakfast regularly is shown to be one of the risk factors of overweight [6-10]. However, the mechanism is still unclear. Furthermore, recent studies indicate that the feeding time regulates, in no small part, *de novo* lipogenesis (DNL), which is a major contributor to fatty liver and visceral obesity in mice [31,32]. Similarly, the relationship between irregular fasting and DNL as well as its linkage to obesity-related phenotypes is still unclear.

To look into the effect of skipping meal, especially at the beginning of the active phase, we conducted early nocturnal fasting (ENF) and then investigated body weight gain, food intake, core body temperature, lipid metabolism and clock-related gene expression in mice. We assessed the relationship between the peripheral clock and DNL under an ENF condition using Gas Chromatography Mass Spectrometer (GC-MS)-based stable isotopic metabolic flux analysis and gene expression analysis.

## Results

### Influence of ENF on physiological variables

**Figure 1:**
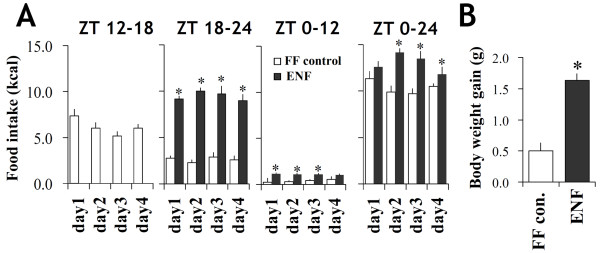
**Effect of ENF on physiological variables.** (**A**) Cumulative food intake during ZT 12–18, ZT 18–24, and ZT 0–12 and daily intake (ZT 0–24). The food intake was obtained by subtracting the amount of food remaining at the end of each term from the amount offered at the beginning of each term. (**B**) Body weight gain during a period of 4 consecutive days, with or without ENF. The body weight gain was calculated from the body weight values before (day 0) and after treatment (day 4). The body weights were measured at ZT 12 on day 4. All of the values are expressed as the mean +/- SEM (n = 6). *p < 0.05 for the ENF group compared with the FF-control group.

We first investigated the physiological effects of ENF on mice that were given *ad libitum* access to food, except short-term restriction from ZT 12 to 18 for 4 consecutive days. Free feeding control (FF-control) mice consumed approximately 65% of their daily intake during the first half of the nocturnal period (ZT 12 to 18) and consumed over 90% throughout the entire nocturnal period (Figure 1A). However, ENF mice consumed approximately 73% of their daily intake during the last half of the nocturnal period (ZT 18 to 24) and still had a significant amount of intake during the diurnal period (Figure 1A). As a consequence, ENF mice significantly increased their total daily intake as compared with the FF-control mice (p < 0.05) (Figure 1A, right panel). ENF mice also exhibited increases in body weight at the end of the experiment (p < 0.05)(ZT 12 on day 4).

**Figure 2:**
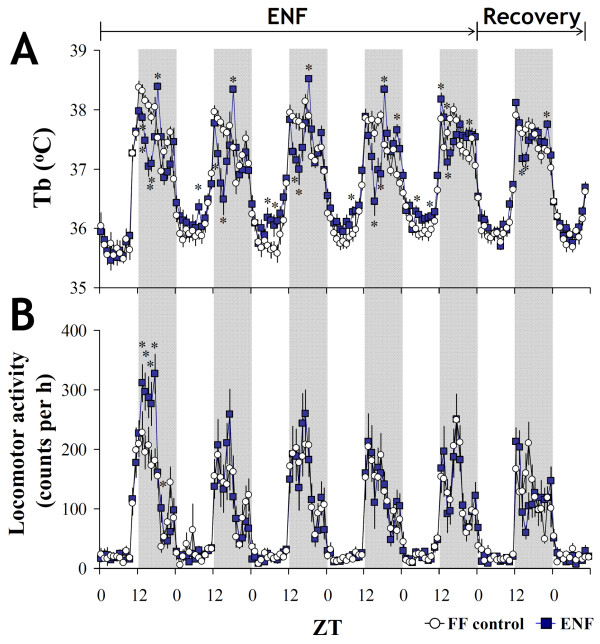
**Effects of ENF on the core body temperature and the locomotor activity.** (**A**) Daily variations in the core body temperature (Tb) and (**B**) the locomotor activity in the control and ENF mice. Dark periods are indicated by the gray area. The data are represented as the mean +/- SEM (n = 6). *p < 0.05 for the ENF group compared with the FF-control group.

We also investigated the influences of ENF on the core body temperature (Tb) and locomotor activity. In the FF-control mice, Tb exhibited clear circadian rhythms, with a rapid rise at ZT 12 that lasted through the nocturnal period (Figure 2A). However, ENF notably reduced Tb in the first half of the nocturnal period and increased Tb during the diurnal period. This different pattern of Tb was partly recovered after switching the dietary conditions from ENF to normal conditions on day 5 (Figure 2A). Interestingly, ENF mice showed increased locomotor activity only on day 1 (p < 0.05), probably because of exploratory behavior, but this activity almost disappeared during the following 4 days (Figure 2B).

### Metabolic value of ENF under isocaloric conditions

**Figure 3:**
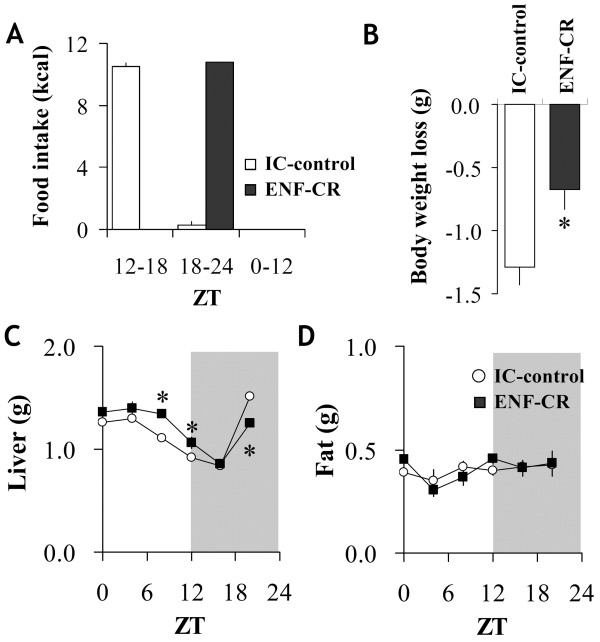
**Effect of ENF on physiologic variables under isocaloric conditions.** (**A**) Cumulative food intake during ZT 12–18, ZT 18–24 and ZT 0–12. C57B/6 mice were fed with a certain amount of chow, which was restricted to 90% of their basal consumption, for 4 consecutive days (10.8 kcal per day). The food was provided at ZT 12 (IC-control) or ZT 18 (ENF-CR) each day. The amount of food intake was obtained by the subtraction of the amount of food remaining at the end of the term from the amount offered at the beginning of each term. (**B**) Body weight loss during the consecutive 4 days, when treated with either IC-control or ENF-CR. The body weight loss was calculated from the body weight values before (day 0) and after (day 4) treatment. (**C**) Liver and (**D**) epididymal fat weights. Tissue weights were measured at ZT 0, 4, 8, 12, 16 and 20 on day 4. Dark periods are indicated by the gray area. All of the values are expressed as the mean +/- SEM (n = 5 or n = 6 per time point). *p < 0.05 for the ENF-CR group compared with the IC-control.

To eliminate the influence of increased food intake by ENF on metabolism, we next tested nocturnal fasting under isocaloric conditions by mild calorie restriction. Calorie restricted ENF (ENF-CR) mice were then compared with isocaloric controls (IC-control). This comparison involved feeding C57B/6 mice a specific amount of food (10.8 kcal per day; 90% of their basal consumption in ENF condition) for 4 consecutive days. The IC-control mice were given the food at ZT 12, while the ENF-CR mice were given the diet at ZT 18. In the IC-control mice, food intake during the first half of the nocturnal period reached over 97% of the daily intake on day 4 (Figure 3A). The IC-control mice in this study appeared to be almost “late nocturnal fasting” because they were assumed to be essentially fasting during the last half of the nocturnal period and the following diurnal period (ZT 18-24 and 0-12) (Figure 3A). The ENF-CR mice consumed almost 100% of their daily intake during the last half of the nocturnal period on day 4. Thus, the lengths of fasting time were approximately equal between the two groups of mice, and the only difference was the timing of taking the meal. Under this regimen, both groups of mice lost body weight because of the CR, whereas the weight loss of the ENF-CR mice was significantly decreased compared with that of the IC-control mice (p < 0.05) (Figure 3B). The ENF mice, moreover, had significantly increased liver weight during the diurnal period and decreased at ZT20 (p < 0.05), but they did not have altered epididymal fat pad weight compared with the IC-control mice (Figure 3C, D).

### Effect of ENF on hepatic lipid metabolism

**Figure 4:**
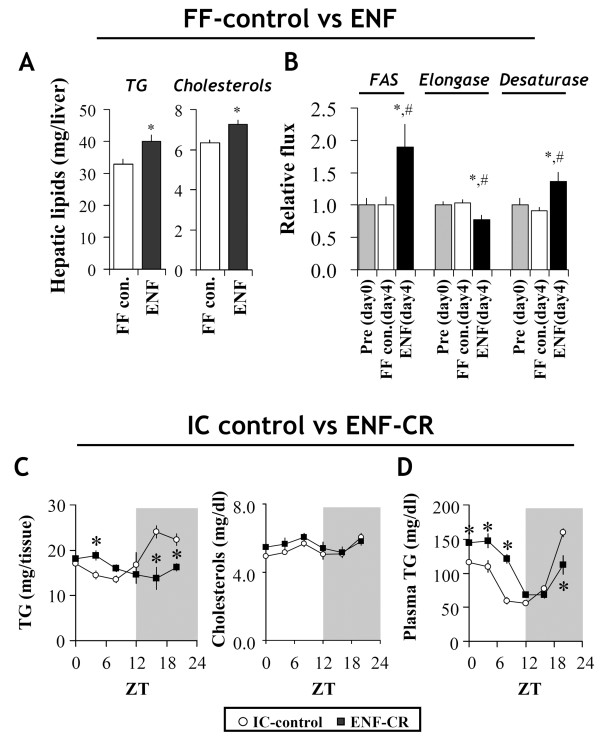
**Effects of ENF on hepatic lipids.** (**A**) Tissue samples were obtained at ZT 12 from C57B6 mice after 4 days under different dietary conditions. Hepatic triglyceride and cholesterol levels were enzymatically determined, as described in the Materials and Methods section. (**B**) The hepatic DNL fluxes before treatment (day 0) and after 4 days under different dietary conditions were determined using deuterated water labeling and the following mass isotopomer distribution analysis. The relative flux of fatty acid synthase (FAS) was estimated from the fraction of newly synthesized palmitate. Elongase and desaturase were assessed as described in the Materials and Methods section. All of the values are expressed as the mean +/- SEM (n = 6). *p < 0.05 for the treatment groups compared with the pre-condition (day 0). #*P* < 0.05 for the ENF compared with the FF-control. (**C**) Circadian variation in hepatic triglycerides (TG) and cholesterols and (**D**) plasma triglycerides (TG) under isocaloric conditions. Dark periods are indicated by the gray area. All of the values are expressed as the mean +/- SEM (n = 6 per timepoint). *p < 0.05 for ENF-CR groups compared with the IC-control.

To understand the rhythmic changes in the liver weight caused by ENF under both the non-IC conditions (shown in Figure 1) and IC conditions (Figure 3C), we correlated the hepatic lipid content and *de novo* lipogenesis (DNL) fluxes using GC-MS. Under non-IC conditions, ENF significantly increased hepatic triglyceride (TG) and cholesterol levels compared with FF-controls at ZT12 after 4 days of treatment (p < 0.05) (Figure 4A). Furthermore, a stable isotopic flux analysis clearly revealed that ENF dramatically enhanced the hepatic DNL flux of fatty acid synthesis and desaturation compared with the FF-control (p < 0.05) (Figure 4B).

Under the IC condition, ENF-CR lowered the hepatic TG levels during the nocturnal period (ZT16, 20) and elevated them diurnal period (ZT4) (p < 0.05), whereas the hepatic cholesterol levels showed no significant difference (Figure 4C). ENF-CR also showed the peak shift of plasma TG levels increasing them during the diurnal period (ZT0, 4, 8) (p < 0.05) compared with the IC-control (Figure 4D).

### Influence of ENF on peripheral lipogenic and clock-related genes

**Figure 5:**
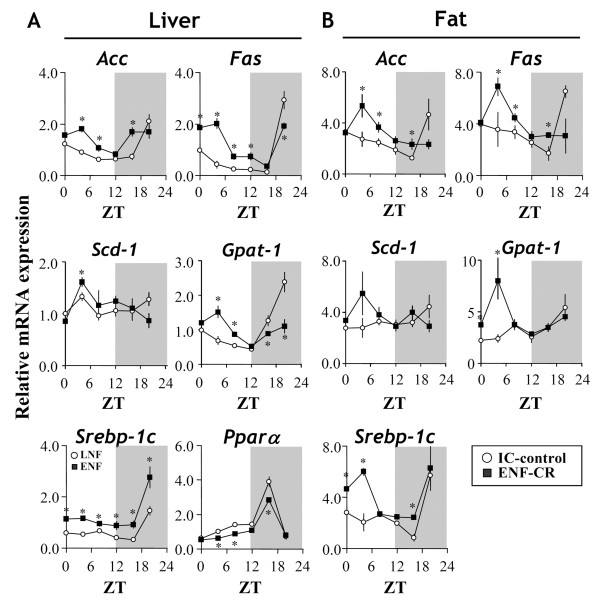
**Circadian alterations in lipogenic genes by ENF under isocaloric conditions.** Liver and epididymal fat samples were collected at ZT 0, 4, 8, 12, 16 and 20 after 4 days of treatment. Gene expression of sterol regulatory element binding protein-1c (*Srebp-1c*), peroxisome proliferator-activated receptor *α* (*Ppar α*), fatty acid synthase (*Fas*), acetyl-CoA carboxylase (*Acc*)*,* stearoyl-coenzyme A desaturase-1 (*Scd-1*) and glycerol-phosphate acyl-transferase-1 (*Gpat-1*) under CR diets (shown in Figure 4) was estimated by RT-PCR under different dietary conditions. The relative levels (IC control = 1) were further normalized to the 18S RNA levels. Dark periods are indicated by the gray area. All of the values are expressed as the mean +/- SEM (n = 6 per time point). *p < 0.05 for the ENF groups compared with the IC-control.

Circadian changes in lipogenic genes were determined in both ENF-CR mice and IC-controls on day 4. In this study, the expression of *Srebp-1c*, *Fas*, *Acc*, *Scd-1* and *Gpat-1* were determined in both the liver and adipose tissues. *Pparα* was also determined in the liver only. As shown in Figure 5A, ENF-CR significantly enhanced the expression of the lipogenic genes *Srebp-1c* at all the time-points. The expressions of *Acc* were enhanced at most of the time-points with the significant difference at ZT4, 8, 16. The expressions of *Fas* were enhanced through ZT0 to 12 and decreased at ZT20. *Gpat-1* expressions, similar to *Fas*, were enhanced in diurnal period (ZT4, 8) and decreased at nocturnal period (ZT16, 20). ENF-CR significantly changed *Scd-1* expression at ZT4 only (increase). *Pparα* expression compared with the IC-control were generally decreased with significant change at ZT4, 8, 16. (p < 0.05) (Figure 5A).

In fat tissue, ENF-CR also significantly increased the expressions of *Srebp-1c* and *Gpat-1* at ZT0, 4, 16(*Srebp-1c* only) without any significant decrease at other points compared to IC-control. The expressions of *Fas* and *Acc* were similar and increased mid-diurnal period to early nocturnal period (ZT4, 8, 16). (p < 0.05).

**Figure 6:**
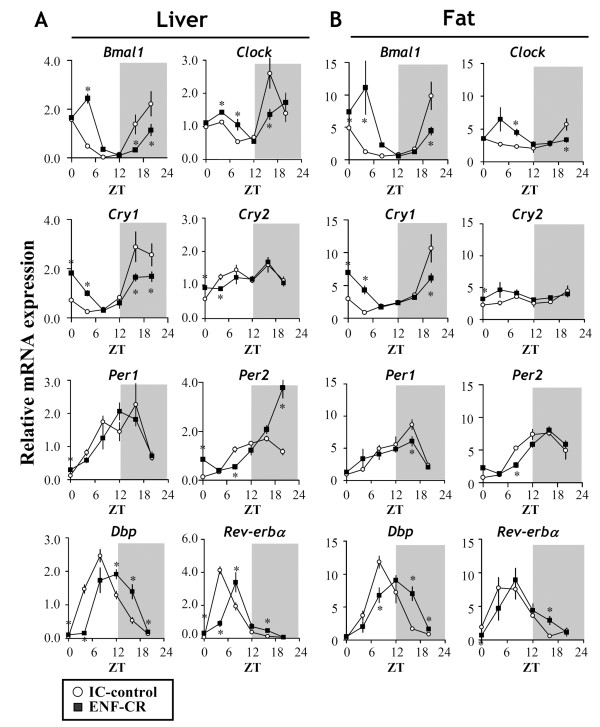
**Circadian alterations in the clock-related genes by ENF under isocaloric conditions.** Liver and epididymal fat samples were collected at ZT 0, 4, 8, 12, 16 and 20 after 4 days of treatment. The gene expression levels of brain and muscle arnt-like protein-1 (*Bmal1*), circadian locomotor output cycles kaput (*Clock*), period1 (*Per1*), period2 (*Per*2), cryptochrome-1 (*Cry1*)*,* cryptochrome-2 (*Cry2*)*,* D-site of albumin promoter binding protein (*Dpp*) and *Rev-erba* under isocaloric diets were estimated by RT-PCR. The relative levels (IC control = 1) were further normalized to 18S RNA levels. The values are expressed as the mean +/- SEM (n = 6 per time point).

**Figure 7:**
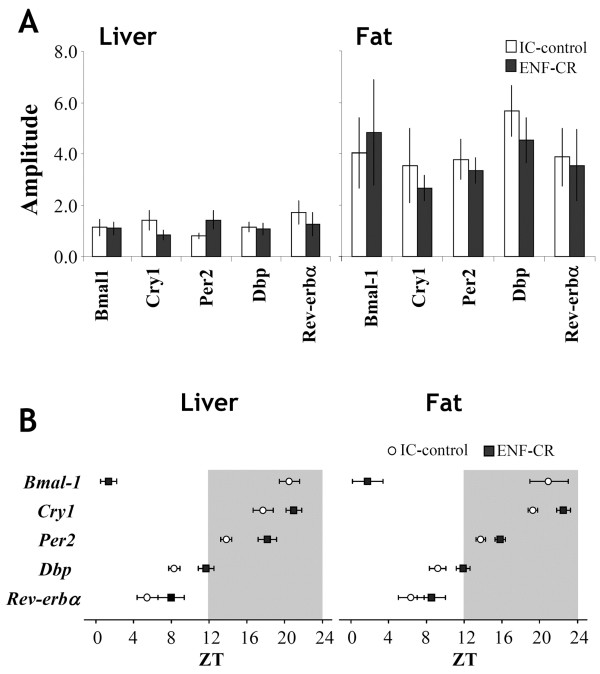
**Effect of ENF on the amplitude and acrophase of clock genes under isocaloric conditions.** (**A**) Amplitudes (the differences between the peaks and troughs of the fitted cosines) and (**B**) acrophases (the times of the peaks in the fitted cosines) in the given gene expression were calculated from cosinor analysis, in which the fit of a 24-h cosine to all of the data was generated by least-squares linear regression. Dark periods are indicated by the gray area. The values are expressed as the mean +/- SEM (n = 6 per time point).

To examine whether ENF alters the peripheral clock, we investigated the expression of *Clock*, *Bmal1*, *Per1*, *Per2*, *Cry1*, *Cry2*, *Dbp* and *Rev-erab α* as representatives of the clock genes. In the liver and fat tissues, ENF-CR increased the expression of *Bmal1*, *Cry1* and *Clock* during the diurnal period and lowered their expression during the nocturnal period compared with the IC-control (Figure 6A). In contrast, ENF-CR increased the expressions of *Per2* in the liver and *Dbp* in both the liver and fat during the nocturnal period (Figure 6A, B). There was no clear change observed in the levels of *Per2* gene expression between ENF and the IC-control in fat tissue (Figure 6A, B). The amplitude and acrophase time of these clock genes were also compared between the groups. As shown in Figure 7A, there were no clear alterations observed in terms of the amplitude of the expression of these genes. In contrast, the acrophase time of most of the genes was drastically changed between the two groups, especially in *Bmal1* (Figure 7B).

## Discussion

This study demonstrated that ENF affects lipid metabolism and the peripheral clocks of mice. The current data clearly show that short-term ENF increases calorie intake and alters the rhythmicity of lipid metabolism, increasing lipogenesis at the levels of transcriptional and metabolic flux. The expressional pattern of clock genes such as *Clock*, *Bmal1*, *Cry1* and *Per2* in both the liver and fat are also altered by ENF. Furthermore, these results are related to weight gain and indicates the importance of having meal at the beginning of active phase to prevent obesity.

Previous studies have shown that irregularly scheduled feeding can alter the biological rhythms independent of lighting conditions [25,33]. Although the SCN clock is mainly regulated by the light/dark cycle, peripheral clock oscillators are affected by daily feeding cycles [23,24]. Time-restricted feeding has also been reported to modify the activities of metabolic enzymes that participate in carbohydrate and lipid metabolic pathways [34,35]. Therefore, the food intake schedule is considered to be the dominant zeitgeber for peripheral circadian oscillators and could be an important factor in regulating metabolism. In this study, ENF in mice caused increased daily food intake compared with unrestricted conditions, whereas locomotor activity remained unchanged. The timing of the main meal was delayed approximately 6 hours by ENF treatment, considering that the mice that were fed *ad libitum* started to eat immediately after the beginning of the dark period (ZT12). Mice that were subjected to ENF without calorie adjustment still had a significant amount of food intake during the diurnal period if they were allowed to eat voluntarily, which could also be considered to be an extension of the feeding time into the diurnal resting period.

ENF without calorie restriction gained more bodyweight during the 4 days compared to FF-control. Their body weights were measured at ZT12, at when little difference was seen in the apparent gastric contents between the two groups though we cannot completely deny the possibility that contents in the intestinal tract affected the body weight. Furthermore, ENF mice ate more than FF-control and body weight gain possibly reflected the increased calorie intake. Thus, we conducted the second experiment under calorie-restricted condition. Body weights in the 2nd experiment were measured at around 1 hr after feeding end in both groups to minimize variation of digestinal state. Nevertheless ENF-CR mice gained more weight compared to IC-control. This result shows 4-day ENF caused increased bodyweight.

In mice and other nocturnal species, Tb exhibits rhythmicity, with the highest temperatures during the dark period and the lowest temperatures during the light period [14]. Our data clearly demonstrated this trend in FF-control mice, for which the Tb was higher during the dark periods and lower in the light periods. The ENF mice, however, showed a remarkably decreased Tb in the first half of the dark period and an increased Tb during the light period compared with control mice. This difference resulted in a reduction of its amplitude. Interestingly, the alteration in Tb was not accompanied by changes in locomotor activity except on the first day, which was assumed to be increased food-exploring behavior. Previous reports indicate that the circadian rhythm of locomotor activity, Tb, and the sleep-wake cycle share a common circadian pacemaker within the SCN [2]. The control of the Tb rhythmicity appears in a human study that showed that dietary-induced thermogenesis varies according to mealtime without altering physical activities [3]. As mentioned above, some features of these rhythmic modalities are affected by exogenous cues other than the photoperiod, such as the feeding time; however, the feeding time does not have complete control of these features. For example, in this study, the Tb of the ENF mice not only delayed its peak points but also decreased the amplitude of its circadian rhythm, whereas the locomotor activities of the ENF mice fundamentally showed no alteration compared with the FF mice. Thus, the dissociation of physiological and behavioral parameters, including Tb and physical activity, which could be controlled in coordination with each other, seems to occur by ENF, at least in mice.

In humans and rodents, *de novo* lipogenesis is known to exhibit a circadian rhythm [36,37]. In general, lipogenesis increases after meals in both humans and nocturnal rodents, and in nocturnal rodents, lipogenesis increases during dark periods (active phases) and decreases during light periods (resting phases) [13,37]. The lipogenic flux has also been reported to increase in response to the nutritional composition, especially with fructose-containing foods, and constantly higher levels are reported in both obese human patients and rodent models [38]. ENF in mice altered hepatic and serum triglyceride levels with increased fatty acid synthesis and desaturation fluxes. Though contents of liver vary during a day, the metabolic fluxes are derived from the values which reflect the accumulation of consecutive 4-day treatment. Thus, these results suggest that ENF switches the metabolism toward lipid deposition with increased lipogenesis in the resting periods, during which energy consumption is low. Because the 4-day treatment used in this experiment was possibly not sufficient to alter epididymal fat weight or to promote the subsequent development of obesity, further study of a much longer treatment regime must be conducted in the future.

Because hepatic expression of lipogenic genes is known to show circadian variation [34,39], we investigated the influence of ENF with isocaloric control (IC-control) on the transcriptional rhythms of clock genes. ENF enhanced hepatic *Srebp-1c*, a master regulator of lipogenesis, and repressed *Pparα;* both of these effects occurred without any shift in the circadian peak from those of the control. ENF strongly affected other downstream genes, specifically *Fas*, *Acc* and *Gpat1*, shifting their peak time from the end of the nocturnal period to the early diurnal period in both the liver and fat tissues. Thus, one of the causes of increased DNL fluxes in ENF mice appears to be the alteration in the transcription of genes related to lipogenesis, in addition to changes in the their peak time. Decoupling from other aspects of physiological rhythmicity, such as locomotor activity, fractures energy supply-consumption balance and excess energy could be stored as fat The alteration in the Tb rhythm of ENF mice could partly explain this tendency.

Daily restricted feeding is well known to entrain peripheral circadian clocks, including the liver and adipose tissue clocks [22-24]. The liver clock system has been reported to play a critical role in various types of metabolism, such as carbohydrate, protein, lipid, and drug metabolism [40-43]. The activities of enzymes and the endocrine system are related to this metabolism and are also known to be modified by time-restricted feeding [34,44,45]. Therefore, food intake scheduling is considered to be an important factor in the regulation of metabolism and possibly has a dominant influence on peripheral circadian oscillators. In mammals, the core components of the biological clock are the clock genes *Clock*, *Bmal1*, *Pers*, and *Crys*, which drive transcriptional-translational feedback loops with approximate 24-hr rhythmicity and mediate the activities of downstream genes [1]. In this study, ENF differentially affected the expression of hepatic and adipose clock genes; the changes were more significant in the liver than in the adipose tissue. In addition, previous studies have demonstrated the relationship between circadian dysfunction and metabolic abnormalities. *Bmal1*- or *Clock*-mutant mice exhibited impaired glucose metabolism [41]. *Clock*-mutant mice increased their caloric intake and their total body weight relative to wild-type controls and exhibited obesity and metabolic syndrome [46]. Liver-specific *Bmal1*-knockout mice have altered circadian glucose homeostasis and increased fat mass [44]. Though the detail of the molecular mechanisms of interaction between Clock system and genes involved in metabolisms are not explained clearly, recent studies have uncovered the links between them. For example, CLOCK controls plasma TG level by regulating the transcription of microsomal TG transfer protein *via* up-regulating of small heterodimer partner [21]. SREBP and downstream lipogenesis have also been reported to be activated by *Clock* and *Bmal1* and repressed by *Rev-erb α*[47]. Especially, BMAL1 is known to be induced during adipogenesis and regulates adipocyte functions [48]. Thus, in this study, delayed expression of *Bmal1/Rev-erb α* was one of the causes of activated transcription of *SREBP-1c*. BMAL1 also up-regulates *Ppar α*, and *Pparα* restrains the transcriptional activity of BMAL1 [40]. In this study, PPARα could repress transcriptional activity of BMAL1 when its expression was insufficient, and possibly suppressed its own transcription throughout a day. With hormonal response to starvation, such desynchronization of transcriptional activation/repression is considered to be one cause of increased DNL. In *Drosophila*, restricted feeding also drives rhythmic expression of clock-related genes in the fat body, which serves the same function as the liver in mammals [49]. Interestingly, clock-mutated *Drosophila* altered not only their metabolic state but also the timing and amount of food consumed [50]. In the present study, the expression of some lipid-related genes showed no alteration in their peak time or amplitude while others changed. Thus, the influence of the photoperiod-dependent clock on metabolisms differs among metabolic pathways even in the same organ. Considering previous reports and the current data, the extension of DNL in ENF mice appears to be caused by the desynchronization of lipid metabolism from the photoperiod-dependent physiological rhythm. Although peripheral clocks adapt to the altered feeding schedule, regulation by photoperiod-dependent clocks continues [23-25]. Therefore, further study that investigates the correlation between chronic ENF and obesity in mice could be more valuable for understanding the effect of timed-fasting on the development of obesity, and especially for understanding the effects of breakfast skipping in humans.

## Conclusions

We found that ENF caused greater daily food intake, weight gain and hepatic lipid deposition in mice. In addition, ENF affected *de novo* lipid synthesis through the concurrent modulation of peripheral clocks. Although further work is needed to extend these initial findings, our data provide important insight into the function of having meal at the beginning of the active phase and the effect of skipping breakfast in humans.

## Materials and methods

### Ethics statement

All of the studies were reviewed and approved by the Animal Care Committee of Ajinomoto Co., Inc. (2010292).

### Animals

Male 12- to 14-week-old C57B6 mice were obtained from Charles River Laboratory, Inc. (Tokyo, Japan). All of the mice were housed in colony cages maintained on a 12:12-h light and dark cycle with free access to water. The mice were fed with commercial rodent chow CRF-1 (Oriental Yeast, Tokyo, Japan). Operations including tissue sampling during the dark period were performed in a dark room with the support of a dim red light. Measurement of body weight and tissue weights were conducted at ZT 12 in the first set of experiments (stated below) and at 6-8 h after the onset of feeding in the second set of experiments (stated below). Excluding the body weight measurements conducted at day 0 and day 4, no additional handling, including cage-changes, was performed during the 4 days. The liver and epididymal fat were collected for lipid and gene expression analyses under isoflurane treatment. All of the collected tissues were immediately placed in liquid nitrogen and stored at -80°C before analysis.

### Feeding schedule

For the first set of experiments, a sufficient amount of chow was available all day (FF-control) or within 6 h of the early nocturnal (ZT 12-18) fasting (ENF) period (i.e., skipping 6 h of food access during the early night). For the second experiment, the diets were designed to be isocaloric, with moderate energy restriction, and consisted of 90% of the basal food intake of 24-h *ad libitum*-fed mice (10.8 kcal per day). Under this feeding condition, all of the mice ate all of the food that was provided. The food was provided at ZT 12 (Isocaloric control) or ZT 18 (ENF-CR) each day. In both of the ENF groups, the food was removed at ZT 12 the next day, and the mice were given access to the food only during the period from ZT 18 to ZT 12 of the next day, with a 6-h fast during the early nocturnal phase. All of the procedures were conducted for 4 consecutive days.

### Measurements of core Tb and locomotive activity

A series of mice underwent surgery to appropriately position intra-abdominal telemetry transmitters (TA10TA-F20, Primetech, Tokyo, Japan). Under isoflurane anesthesia, a small incision was made in the abdominal cavity, and the transmitters were introduced into the peritoneum. The mice were left to recover for 2 weeks. The transmitters were programmed to collect temperature and activity data every 5 min.

### Real-time PCR

Total RNA was extracted from the homogenized liver and fat tissue using an RNeasy kit (Qiagen, Germantown, MD) following the manufacturer’s instructions. Equal amounts of RNA were reverse-transcribed using an Omniscript RT kit (Qiagen) as per the manufacturer’s instructions. Primers for RT-PCR were designed using the primer design software Primer3, and the sequence homologies of related proteins were checked. The 36B4 and 18S ribosomal RNA were used as endogenous controls. RT-PCR was performed on an ABI Prism® 7900 Sequence Detection System (PE Applied Biosystems, Foster City, CA), and the data obtained were analyzed using the software provided. The reaction mixture consisted of 4 μl of cDNA template, 10 μl of SYBR Green PCR master mix (Roche Biochemicals, IN), and 1 μl of 10 μM forward and reverse primers in a 20-μl reaction volume. The PCR protocol consisted of one 10-min denaturation cycle at 95°C, followed by 40 cycles of denaturation at 95°C for 15 sec and an annealing/extension step at 60°C for 1 min. Standard curves for each gene, in addition to the 36B4 and 18S ribosomal RNA controls, were obtained. All of the RT-PCR data from the liver and adipose tissue were expressed as relative mRNA levels after normalizing to 36B4 and 18S, respectively. The primer pair sequences are listed in Additional file 1: Table S1.

### Tissue triglyceride and total cholesterol analysis

Liver and adipose lipid extractions were performed using isopropanol and hexane, as previously described [51,52]. Briefly, 150 mg of liver or 40 mg of epididymal fat tissue was homogenized in 1.5 ml of isopropanol and then sonicated for 30 min, after the addition of 3 ml of hexane. As an internal standard, 0.1 mg of triheptadecanoic acid was added to each sample. The homogenate was incubated overnight for further extraction. After the addition of 1 ml of phosphate-buffered saline (PBS), the mixture was centrifuged at 2,500 rpm for 30 min. The supernatant was collected in new tubes and was then evaporated until dry.

To analyze the triglyceride fatty acid profile, the triglyceride fraction was saponified with 500 μl of 0.5 N NaOH in methanol at 70°C for 1 hour and then re-esterized with boron trifluoride (BF_3_) in methanol, as described previously [53]. The fatty acid methyl esters were analyzed using electron impact ionization by GC-MS on an Agilent 5975C model coupled with a 7890A model gas chromatograph. A DB-225 MS capillary column (30 m × 0.25 mm film, 0.25 μm Agilent) was used in all of the lipid analyses. The initial oven temperature was 190°C, which was then increased by 3°C/min to 230°C and held for 2 min. The MSD transfer line was set at 280°C. The components of the sample were repetitively scanned from 50 to 550 *m*/*z*. The MS quad temperature was 150°C, and the MS source was set at 230°C.

Peak enumeration and spectral deconvolution were performed by AMDIS software ( http://hemdata.nist.gov/mass-spc/amdis/; National Institute of Standards and Technology). The resulting ELU files were further analyzed by SpectConnect software ( http://spectconnect.mit.edu/) to identify well-conserved peaks among multiple GC-MS chromatograms [54].

For the determination of the tissue lipid content, 100 μl of the hexane phase was evaporated to dryness and dissolved in 100-500 μl of 10% Triton X-100 in a 2-propanol solution. The samples were then sonicated in a bath sonicator for 15 min and vortexed. The amounts of triglyceride and total cholesterol were determined using the Triglyceride and Cholesterol E-tests (Wako Pure Chemical Industry, Osaka, Japan), respectively.

### Determination of lipogenesis flux

Two weeks prior to euthanasia, the mice were given an intraperitoneal injection of deuterated water (D_2_O) that was equal to 4% of their body weight in saline at 10:00 AM. The animals were then maintained on drinking water containing 6% D_2_O for 2 weeks. This procedure was designed to maintain deuterium enrichment in the body water at 3%-4% throughout the study [51,53].

Using D_2_O and mass isotopomer analysis, the rates of synthesis can be calculated from the ratio of isotope-labeled fatty acids to unlabeled fatty acids [53]. The major peaks in the ion chromatogram of fatty acids in the liver are the long-chain fatty acids palmitate (16:0), stearate (18:0) and oleate (18:1). The spectra of the palmitate (270-276 *m*/*z*), stearate (297-304 *m*/*z*) and oleate (263-276 *m*/*z*) peaks were analyzed for their isotopomer distribution and deuterium content, which were used to calculate the fraction of newly synthesized FA, and the fractional synthesis rates of palmitate were used to estimate fatty acid synthase flux. Estimates of chain elongation to stearate and estimates of desaturation to oleate were performed, as previously described [51,52]. All of the fluxes after the dietary treatments (day 4) were normalized to those from pre-treatment (day 0).

### Statistics

The data are presented as the mean +/- SEM unless otherwise indicated. The data were analyzed by an unpaired two-tailed Student’s *t*-test (body weight gain, tissue weight and hepatic lipid contents of single time point, food intake, DNL fluxes) or a two-way ANOVA (time of day × diet), followed by Tukey’s post hoc test (time course data of Tb, locomotor activity, tissue weight, serum or hepatic metabolic parameters, and transcripts) to determine the statistical significance for all of the multiple comparison procedures. P < 0.05 was considered to be statistically significant. A 24-hr-period cosine curve was fitted by the nonlinear least-square method to the time-course expression data of the clock-related genes, as previously described with minor modifications [55].

## Competing interests

The authors declare that they have no competing interests.

## Authors’ contributions

NK designed research; CY, NS, and YN designed and conducted research; CY and SS analyzed the data; and CY wrote the paper. All authors read and approved the final manuscript.

## Supplementary Material

Additional file 1 **Table S1.** Primer sequences used for RT-PCR. Click here for file
