# Supplementary material for: Early nocturnal meal skipping alters the peripheral clock and increases lipogenesis in mice
Source: Nutr Metab (Lond). 2012 Sep 10;9:78. doi: 10.1186/1743-7075-9-78 (PMC3515427; doi:10.1186/1743-7075-9-78)
Supplement: Additional file 1 — Table S1. Primer sequences used for RT-PCR. [file 1743-7075-9-78-S1.doc]

**Table S1:** Primer sequences used for RT-PCR.

| Gene name | Primers | Size (bp) |
| --- | --- | --- |
| *Clock* | TTGCTCCACGGGAATCCTT | 74 |
|  | GGAGGGAAAGTGCTCTGTTGTAG |  |
| *Bmal1* | CCAAGAAAGTATGGACACAGACAAA | 81 |
|  | GCATTCTTGATCCTTCCTTGGT |  |
| *Per2* | ATGCTCGCCATCCACAAGA | 72 |
|  | GCGGAATCGAATGGGAGAAT |  |
| *Cry1* | CTGGCGTGGAAGTCATCGT | 77 |
|  | CTGTCCGCCATTGAGTTCTATG |  |
| *Cry2* | TGTCCCTTCCTGTGTGGAAGA | 67 |
|  | GCTCCCAGCTTGGCTTGA |  |
| *Dbp* | ACAGCAAGCCCAAAGAACC | 67 |
|  | GAGGGCAGAGTTGCCTTG |  |
| *Rorα* | ACCGTGTCCATGGCAGAAC | 61 |
|  | TTTCCAGGTGGGATTTGGAT |  |
| *Rev-erbα* | GGGCACAAGCAACATTACCA | 64 |
|  | CACGTCCCCACACACCTTAC |  |
| *SREBP-1c* | GGAGCCATGGATTGCACATT | 149 |
|  | GCTTCCAGAGAGGAGGCCAG |  |
| *FAS* | CGAACCTGGCTGCCTACTAC | 97 |
|  | AAGGCTACACAGGCTCCAAA |  |
| *ACC* | TGCCCTCAATTCTGTCCACT | 108 |
|  | CGAAGGTTCTCTGCTCCAAG |  |
| *SCD1* | GCGATACACTCTGGTGCTCA | 117 |
|  | CCCAGGGAAACCAGGATATT |  |
| *GPAT1* | AGCAAGTCCTGCGCTATCAT | 72 |
|  | GATTCCCTGCCTGTGTCTGT |  |
